# Semantic Working Memory Predicts Sentence Comprehension Performance: A Case Series Approach

**DOI:** 10.3389/fpsyg.2022.887586

**Published:** 2022-04-29

**Authors:** Autumn Horne, Rachel Zahn, Oscar I. Najera, Randi C. Martin

**Affiliations:** ^1^T.L.L. Temple Neuroplasticity Lab, Department of Psychological Sciences, Rice University, Houston, TX, United States; ^2^Department of Psychology, University of Texas at Austin, Austin, TX, United States

**Keywords:** working memory, sentence comprehension, cognitive neuropsychology, semantic working memory, phonological working memory

## Abstract

Sentence comprehension involves maintaining and continuously integrating linguistic information and, thus, makes demands on working memory (WM). Past research has demonstrated that semantic WM, but not phonological WM, is critical for integrating word meanings across some distance and resolving semantic interference in sentence comprehension. Here, we examined the relation between phonological and semantic WM and the comprehension of center-embedded relative clause sentences, often argued to make heavy demands on WM. Additionally, we examined the relation between phonological and semantic WM and the comprehension of transitive and dative active and passive sentences, which may also draw on WM resources depending on the number of propositions that must be maintained and the difficulty of processing passive clauses. In a large sample of individuals with aphasia (*N* = 56), we assessed whether comprehension performance on more complex vs. simpler active-passive or embedded relative clause sentences would be predicted by semantic but not phonological WM when controlling for single word comprehension. For performance on the active-passive comprehension task, we found that semantic WM, but not phonological WM, predicted comprehension of dative sentences when controlling for comprehension of transitive sentences. We also found that phonological WM, but not semantic WM, predicted mean comprehension for reversible active-passive sentences when controlling for trials with lexical distractors. On the relative clause comprehension task, consistent with prior results, we found that semantic WM, but not phonological WM, predicted comprehension of object relative clause sentences and relative clause sentences with a passive construction. However, both phonological WM and semantic WM predicted mean comprehension across all relative clause types for reversible trials when controlling for trials with lexical distractors. While we found evidence of semantic WM’s role in comprehension, we also observed unpredicted relations between phonological WM and comprehension in some conditions. Post-hoc analyses provided preliminary evidence that phonological WM maintains a backup phonological representation of the sentence that may be accessed when sentence comprehension processing is less efficient. Future work should investigate possible roles that phonological WM may play across sentence types.

## Introduction

Sentence comprehension is a complex cognitive process that requires us to continuously access, maintain, and integrate incoming information. Perhaps then, it is unsurprising that there is mounting evidence that such a complex process is not isolated but rather draws on other cognitive systems to operate successfully (Fedorenko et al., 2006; Baldo and Dronkers, 2007; January et al., 2009). One cognitive system that has been shown to support sentence comprehension is working memory (WM; Martin and He, 2004; Fedorenko et al., 2006; Tan et al., 2017). WM allows listeners or readers to keep verbal representations active over short periods of time so that later parts of the sentence can be integrated with earlier ones. For example, if listeners heard the sentence “The boy who had red hair carried the girl,” they could draw on WM to maintain the sentence’s subject “boy” over the course of the intervening descriptive clause “who had red hair” until it can be integrated with the verb “carried.”

### The Domain-Specific Model of Working Memory

**Figure 1:**
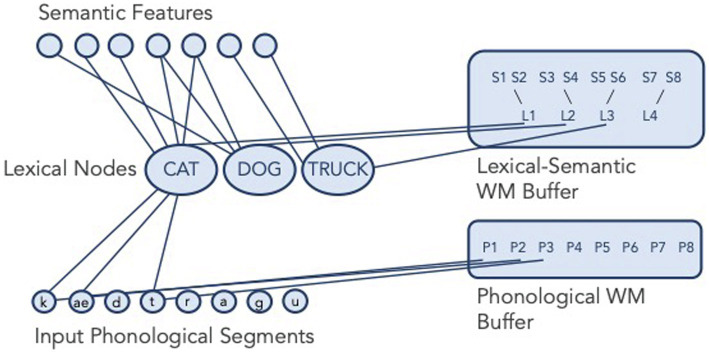
The domain-specific model of WM (adapted from Martin et al., 2021a).

While there are many different conceptualizations of WM, evidence from neuropsychological studies generally favors the domain-specific model of WM (Figure 1; Martin et al., 1999, 2021b). The domain-specific model of WM postulates separate WM buffers for semantic and phonological information that are also separate from long-term knowledge in these domains. Semantic WM maintains semantic representations—the meanings associated with words—and phonological WM maintains phonological representations—the speech sounds associated with words. The domain-specific model is distinguished from embedded processes models of WM (e.g., Cowan et al., 2021) as well as more traditional buffer models of WM (e.g., Baddeley et al., 2021). In contrast to embedded processes models of WM where WM is the activated portion of long-term memory, the domain-specific model cites evidence from people with brain damage that shows that WM can be damaged separately from long-term memory (Martin et al., 1999) to make the claim that WM capacity is separate from long-term memory. In contrast to other buffer models such as Baddeley’s multicomponent model of WM (Baddeley and Hitch, 1974; Baddeley et al., 2021) which has a single verbal WM buffer for phonological information, the domain-specific model includes separate buffers for different types of verbal representations.

Evidence for the domain-specific model of WM comes primarily from work with people who have brain damage resulting in impairment to phonological and/or semantic WM. This work has shown that semantic and phonological WM can be distinguished both behaviorally (e.g., Martin et al., 1994) and neurally (e.g., Martin et al., 2021a). Semantic WM is measured with tasks such as category probe which requires the short-term maintenance of semantic representations. In the category probe task, people hear a list of words followed by a probe word and must indicate whether the probe word is in the same category as any of the words from the list (e.g., list: table, sign, daisy, and bear; probe: rose; Martin et al., 1994). Phonological WM is measured with tasks requiring the short-term maintenance of speech sounds, such as the digit matching task. In the digit matching task, people hear two lists of digits separated by a short pause and are asked to indicate if the two lists are the same or different (e.g., 5 8 7 2 5 7 8 2, response: no; Martin et al., 1994). In general, people with semantic WM deficits perform poorly on the category probe task compared to the digit matching task, and people with phonological WM deficits perform poorly on the digit matching task compared to the category probe task. In addition to the behavioral evidence that semantic and phonological WM capacities are distinct and can be damaged separately, there is neural evidence for distinct localizations of proposed semantic and phonological buffer regions. Of note is a recent multivariate lesion symptom mapping study by Martin et al. (2021a) which found that decrements in phonological WM capacity were associated with damage to the supramarginal gyrus and principally subcortical regions associated with articulation. In contrast, decrements in semantic WM performance were related to damage to voxels in the inferior frontal gyrus and angular gyrus. These findings are aligned with other studies of the cortical regions associated with phonological and semantic WM (Paulesu et al., 1993; Martin et al., 2003; Hamilton et al., 2009; Pisoni et al., 2019; Yue et al., 2019; Yue and Martin, 2021).

### Phonological Working Memory and Sentence Comprehension

Semantic and phonological WM capacities also differ in their role in sentence comprehension. Generally, phonological WM has not been found to be critical for sentence comprehension, but semantic WM has. Support for this claim comes primarily from the sentence comprehension abilities of people with specific impairments to their phonological WM. For example, Butterworth et al. (1986) reported a patient RE, who also had impaired phonological WM but who had unimpaired sentence comprehension, even on sentences with nonstandard embedded clauses. However, RE’s phonological WM deficit was a result of a developmental disorder, so it is possible that she had developed strategies to compensate for restricted phonological WM in situations where comprehension was more demanding. Soon after, Martin (1987) reported a patient, EA, with a phonological WM deficit because of a stroke who, nevertheless, had generally normal relative clause sentence comprehension. In the relative clause sentence comprehension task, EA was asked to listen to sentences and point to the correct picture depicting the sentence. The sentences used were center-embedded relative clause sentences that separated the subject from either its action clause or descriptive clause (Table 1). Thus, EA was required to maintain information about the sentence’s subject over the course of the intervening relative clause to integrate it with the action or descriptive clause. EA showed normal comprehension of all but the most complicated sentences. In the case of sentences with more complicated embedded structures, it is possible that listeners, particularly those with language deficits, have difficulty keeping pace with integrations and lag behind when processing the subsequent input. Having a phonological store to fall back on may become important in such situations, and one might speculate that EA, with her reduced phonological WM capacity, was unable to rely on this backup store.

**Table 1:** Examples of relative clause sentence types, with each type containing an action clause and a descriptive clause (adapted from Martin, 1987).^*^

Sentence type	Action clause	Example
1	Main, active	The boy that had red hair carried the girl
2	Embedded, active (subject relative)	The boy that carried the girl had red hair
3	Main, passive	The boy that had red hair was carried by the girl
4	Embedded, passive	The boy that was carried by the girl had red hair
5	Embedded, active (object relative)	The boy that the girl carried had red hair

* *The table shows whether the action clause was the main or embedded clause and the structure of the action clause. The descriptive clause was always in a transitive active form*.

While EA had difficulty with the most complex embedded relative clause structures, other patients have been reported who had reduced phonological WM capacity but nevertheless were able to perform well on sentence comprehension tasks. Waters et al. (1991) reported a patient BO who had limited phonological WM capacity but good sentence comprehension, including good performance on even quite complicated relative clause sentence comprehension materials (like sentence type 5 in Table 1, on which EA performed poorly) when information was presented auditorily. BO’s performance declined somewhat on a sentence comprehension task with visual presentation, but she performed well on auditory sentence comprehension (which one would assume would put the most demand on phonological WM capacity) and poorly on single word reading. Thus, it is more likely that BO’s poor comprehension with visual presentation is due to her reading difficulties rather than her phonological WM deficit. Gvion and Friedmann (2012) also reported patients with phonological WM deficits who were able to comprehend complex relative clause sentences and, interestingly, other types of complex sentences such as garden path sentences (Friedmann and Gvion, 2007). However, all the previously described neuropsychological work focused on the intact sentence comprehension abilities of people with deficits to phonological WM. They did not make a distinction between phonological and semantic WM or measure patients’ semantic WM ability to understand its relationship with sentence comprehension.

### Semantic Working Memory and Sentence Comprehension

Other work has compared the sentence comprehension of people with phonological WM deficits and those with semantic WM deficits. Martin and Romani (1994) reported that people with semantic WM deficits performed poorly on an attribute judgment sentence comprehension task as the number of word meanings to be maintained increased. In the attribute judgment sentence comprehension task, people with semantic WM deficits had more difficulty answering the question “Which is quiet, a concert or a library?” compared to the question “Is a library quiet?” In the first question, there are more semantic representations that must be maintained to answer the question correctly, making comprehension more difficult for people with restricted semantic WM capacities. Additionally, Martin and Romani (1994) and Martin and He (2004) reported that people with semantic WM deficits had more difficulty detecting anomalies in sentences compared to people with phonological WM deficits when integration of adjectives with nouns or nouns with verbs was delayed rather than immediate. For example, they did poorly when the nouns came before the verb (e.g., “The rugs, mirrors, and vases cracked during the move”) and integration of the first noun with the verb was delayed until after processing the intervening nouns. They did much better when the nouns came after the verb such that each noun could be integrated immediately as the object of the verb as it was heard (e.g., “The movers cracked the vases, mirrors, and rugs”). Martin and colleagues explained these findings on the grounds that once the meanings of words were integrated into propositions, their maintenance did not depend on semantic WM.

Semantic WM has also been related to the ability to resolve interference during sentence comprehension. Tan and Martin (2018) tested a group of nine individuals with left hemisphere brain damage on measures of semantic WM, phonological WM, and sentence comprehension. In the sentence comprehension task, they manipulated levels of semantic and syntactic interference. These sentence manipulations were based on the cue-based parsing approach to comprehension which hypothesizes that interference in comprehension is caused when there is competition at integration. For example, when hearing the sentence, “The package from Maria arrives tomorrow,” the listener would generate semantic and syntactic features associated with each word in the sentence (Lewis et al., 2006). For example, “package” would be associated with syntactic features such “noun,” “singular,” and “subject” as well as semantic features such as “inanimate” and “can be mailed.” When a later word in the sentence requires integration with an earlier word, retrieval cues for locating the earlier word are generated. In this sentence, retrieval cues would be generated when processing the verb “arrives” which would specify that the verb should be associated with a word in the sentence with grammatical features including “noun” and “subject,” and semantic features such as “can arrive.” The word in the sentence whose features match up most closely with the retrieval cues is ultimately integrated (in this case “package”). Interference arises from other nouns that have partial overlap with the features associated with the retrieval cues (i.e., Maria is a noun and can arrive but is not a subject). Tan and Martin (2018) manipulated semantic interference based on whether a noun in a dependent clause was semantically plausible or implausible as the subject of the main verb (Table 2). It should be noted that it is not the semantic similarity of the nouns to each other that is important (in this example, Maria and package do not share semantic features), but rather both have semantic features that make them plausible as entities that can serves as subjects of the verb “arrive” (e.g., they move through space). Syntactic interference was manipulated based on whether a noun in the dependent clause was a grammatical subject or not (i.e., direct object in the examples in Table 2). They found that the semantic WM capacities of the patients, but not phonological WM capacities, predicted the ability to resolve semantic interference. Neither semantic nor phonological WM capacities predicted the ability to resolve syntactic interference. These findings have been corroborated by a similar study testing the semantic WM, phonological WM, and sentence comprehension abilities of a large group of healthy undergraduates. In this sample as well, semantic WM, but not phonological WM, predicted the ability to resolve semantic interference in sentence comprehension (Tan et al., 2017).

**Table 2:** Examples of stimuli with syntactic and semantic interference (adapted from Tan and Martin, 2018).^*^

Sentence type	Example
Low syntactic/Low semantic	The jockey who had challenged the unbeatable *record* yesterday will win
Low syntactic/High semantic	The jockey who had challenged the unbeatable *champion* yesterday will win
High syntactic/Low semantic	The jockey who claimed that the *record* was unbeatable yesterday will win
High syntactic/High semantic	The jockey who claimed that the *champion* was unbeatable yesterday will win
**Question**	*Will the jockey win?*

* *Embedded noun with interfering semantic and/or syntactic features is italicized*.

The previously described studies (e.g., Martin and Romani, 1994; Martin et al., 1994; Martin and He, 2004; Tan et al., 2017; Tan and Martin, 2018) highlight the role of semantic WM in supporting comprehension when integration is delayed and when there is semantic interference. These two principles could explain the role that semantic WM may play in the comprehension of other complex sentence structures, such as sentences with object relative structures (see Table 1, type 5). For example, in the object relative sentence, “The boy that the girl pushed had red hair,” integration of the main clause subject “boy” with the main clause verb and the descriptive clause “had red hair,” are both delayed. There is also semantic interference in this object relative clause sentence because the listener is required to retrieve a noun that is a subject of “had red hair,” and both the main clause subject “boy” and the relative clause subject “girl” are semantically plausible nouns. Thus, for the relative clause sentences, we predicted that semantic WM, but not phonological WM, would predict performance on sentence types with greater WM demands due to long distance dependencies and greater interference (e.g., object relatives) when contrasted with performance on sentence types with fewer WM demands (e.g., subject relatives).^1^

While relative clause sentences are most often used to investigate the WM demands of sentence comprehension, previous work has also demonstrated the role of WM in the comprehension of simpler sentence constructions. Even when long distance dependencies across intervening material are not present, past work has still demonstrated a role for WM in comprehension, for instance, in simple transitive passive sentences (Pettigrew and Hillis, 2014). Why might WM play a role in comprehension of simple passive sentences? It is possible that an expectation of the first noun as agent is generated for all sentences, but then, this assumption must be overridden when the passive morphology is processed (Ferreira et al., 2002; also see Traxler et al., 2002 for evidence of this revision process in relative clause sentence processing and Novick et al., 2009 for evidence from garden path sentence processing with reduced relatives). One might posit that re-interpreting the initial assignment draws on some WM capacity, potentially with semantic WM involved in holding on to the semantics of the initial noun while re-interpreting its role assignment. It is also possible that while this re-assignment is made, subsequent information must be held in a phonological form until attention can be directed toward it, thus requiring phonological WM. Also, because dative structures, which were included in our active-passive comprehension task, require determining the role assignment of three nouns relative to two in the transitive sentences (Table 3), WM would be more heavily taxed to maintain the larger number of propositions in the dative sentences, particularly for those with lower WM spans (Caplan and Waters, 1999).

**Table 3:** Examples of active-passive sentence types (adapted from Martin, 1987).

Sentence type	Example
Transitive active	The boy pulled the girl
Transitive passive	The girl was pulled by the boy
Dative active	The boy read a book to the girl
Dative passive	The book was read to the girl by the boy

For the relative clause comprehension task, the first contrast of interest was comprehension for object relative (type 5, Table 1) vs. subject relative (type 2, Table 1) clauses. The motivation for this contrast is described above. This contrast has been common in the literature because the two sentence types are closely matched except for the word order in the embedded clause (e.g., Traxler et al., 2002). Three other contrasts were also assessed. One was between sentences that included a passive clause (types 3 and 4, Table 1) vs. sentences that included an active clause (types 1 and 2. Table 1). Because passive forms may require the revision of the initial noun’s thematic role with respect to the verb (Bever, 1970; Ferreira et al., 2002), one might expect greater WM demands for sentences including passives. Another contrast that we assessed was between sentences with a passive in the embedded clause (type 4, Table 1) vs. sentences with a passive in the main clause (type 3, Table 1). Processing embedded clauses is cognitively demanding because it simultaneously involves maintaining preceding representations in the main clause subject while processing the embedded clause (e.g., Barry and Lazarte, 1995). Thus, processing an embedded passive clause should be more demanding than processing a main clause passive. Finally, we contrasted mean performance on all five types of sentences for the trials tapping into syntactic processing vs. trials tapping into the maintenance of lexical representations. In both trial types, participants were asked to choose the picture that matched the sentence they just heard. In the trials tapping into syntactic processing (referred to as “reversal trials”), the distractor picture depicted a reversal of either the descriptive clause or action clause. In the trials tapping the maintenance of lexical representations (referred to as “lexical distractor trials”), the distractor picture depicted the incorrect noun, verb, or adjective. This contrast is intended to reveal WM’s relation to syntactic analysis vs. simple maintenance of lexical information.

Because processing passive constructions has been argued to be more capacity demanding than processing of actives, we also wanted to assess patients’ comprehension of simpler transitive and dative sentences with active and passive structures. Examples of these sentences are shown in Table 3. In the active-passive comprehension task, we tested three contrasts. The first was comprehension of dative vs. transitive structures. The second contrast was mean comprehension of sentences with both the dative and transitive structures that included a passive clause vs. mean comprehension for sentences with both dative and transitive structures that included only active clauses. Finally, we contrasted mean performance on all four sentence types that tapped into syntactic processing with reversible distractor pictures vs. mean performance on trials with lexical distractors that did not require syntactic processing.

### Current Study

In the current study, we revisit the relationship between sentence comprehension and WM, specifically phonological and semantic WM, in a large sample of people with left hemisphere brain damage resulting in WM deficits. To do this, we tested 56 people with left hemisphere brain damage on measures of sentence comprehension, semantic WM, phonological WM, and single word semantic and phonological processing. While many studies of sentence processing have specifically investigated performance in people with agrammatic Broca’s aphasia (e.g., Thompson et al., 1999; Garraffa and Grillo, 2008; Grillo, 2009), others have shown that the same patterns of comprehension performance apply across a range of clinical classifications (Naeser et al., 1987; Caplan and Waters, 1999; Martini et al., 2019). Thus, our sample included people from different clinical subtypes of aphasia, with 12/56 falling into the classification of Broca’s aphasia according to the Western Aphasia Battery (WAB; Kertesz, 1982) and 5/56 showing evidence of agrammatism^2^ (Supplementary Table 1). Based on previous neuropsychological findings (Martin and Romani, 1994; Martin and He, 2004; Tan and Martin, 2018), we predicted that semantic WM, but not phonological WM, would predict performance on sentence comprehension after controlling for single word processing. We controlled for each participant’s single word processing because performance on the sentence comprehension and WM tasks required participants to process the semantic and/or phonological information associated with individual words. Thus, we used a case series approach and ensured that any deficits in performance could not be accounted for by single word phonological or semantic processing deficits rather than WM deficits. While others have investigated the relation between sentence comprehension and WM (Martin, 1987; Waters et al., 1991; Gvion and Friedmann, 2012; Pettigrew and Hillis, 2014; Varkanitsa and Caplan, 2018), we are the first to do so while also distinguishing between semantic and phonological domains of verbal WM (Martin et al., 1999, 2021a) and controlling for the role of single word processing abilities in sentence comprehension rather than using these measures to screen for high versus low performers on sentence comprehension (Caplan et al., 2007).

## Materials and Methods

### Participants

Participants were 56 people with chronic aphasia tested in the T.L.L. Temple Neuroplasticity Lab at Rice University. The mean participant age was 62.46 years (SD = 14.07, range = 22–85), and the mean education level was 16.24 (SD = 2.49; range = 11–22) years. Twenty-two participants were female, and forty-four were right-handed. Descriptive statistics for performance on WM and single word processing measures can be found in Table 4, and descriptive statistics for sentence comprehension performance are in Tables 5, 7. Individual patient performance on all measures is shown in Supplementary Table 2. The tasks were administered to participants as they were enrolled in studies in the laboratory, from 2005 to 2020. It took approximately 4–6 h for participants to complete all tasks. All participants were tested in accordance with Rice University’s Institutional Review Board protocol FY2016-170.

**Table 4:** Descriptive statistics for participants’ behavioral task performance (*N* = 53).

	Mean	Standard deviation	Range
Category probe	2.40	1.38	0.42–6.5
Digit Matching span	4.08	1.38	0.56–6.5
Digit span	3.87	1.46	1–8.5
Semantic d’	2.91	0.65	1.60–4.14
Phonological d’	3.58	0.49	1.74–4.14
Pyramids and palm trees	90%	11%	42–100%
Consonant discrimination	85%	11%	52–98%
Auditory lexical decision	79%	11%	52–98%

**Table 5:** Descriptive statistics for performance on each trial type in the active-passive sentence comprehension task (*N* = 56).

	Mean	Standard deviation	Range
Transitive active	0.81	0.19	0.38–1
Transitive passive	0.70	0.24	0.25–1
Dative active	0.86	0.17	0.38–1
Dative passive	0.74	0.24	0.13–1
Lexical substitutions	0.92	0.09	0.55–1
**Combined conditions**			
Dative mean	0.80	0.17	0.38–1
Transitive Mean	0.76	0.19	0.38–1
Active Mean	0.83	0.16	0.44–1
Passive mean	0.72	0.22	0.32–1

### Phonological Working Memory

#### Digit Span

Digit span was measured using the Digit Span Forward subtest from the WAIS-R (Wechsler, 1981). Participants repeated back auditorily presented list of digits, ranging in length from 1 to 9 items, with 2 lists at each list length. The task began with the repetition of one-item lists and proceeded to longer list lengths until the participant missed both lists at a given list length. Span was calculated as the last length in which a participant got both lists correct plus 0.5 for any additional lists correct.

#### Digit Matching Span

Digit matching span was used as an additional measure of phonological WM which was unique in that it required only minimal speech output (Martin et al., 1994). In the digit matching task, participants heard two lists of digits separated by a 2000 ms pause. They were asked to indicate with a key press or a single word response (“yes” or “no”) if the two lists were the same or different. The task began with two-item lists and proceeded to longer list lengths to a maximum of six-item lists, until participants scored less than 75% correct at a given list length. There were 24 trials at each list length. Span was calculated as the estimated list length at which they would score 75%, using linear interpolation between the lists spanning 75% percent correct. For example, if a participant scored 90% correct on two-item lists and 60% correct on three-item lists, their interpolated span for 75% correct would be 2.5 items [i.e., 2 + (75–60)/(90–60)]. The digit span and digit matching task are assumed to measure phonological WM because random lists of digits carry little semantic information, and thus, maintenance depends primarily on retention of phonological information.

### Semantic Working Memory

#### Category Probe

Participants were presented with lists of items followed by a probe word and were asked to indicate if the probe word was in the same category as any of the items from the lists (Martin et al., 1994). For example, if the list was “rose, table, dog” and the probe word was “chair.” The correct response would be “yes” because table and chair are in the same category of furniture. Semantic categories in the task included animals, body parts, clothing, flowers, trees, fruits, insects, weather, and kitchen equipment. The lists started at two items and increased to a maximum of seven items until performance dropped below 75% correct. There were 20–24 trials at each list length. The dependent variable was the list length at which they scored 75% correct, calculated by linear interpolation.

### Phonological Processing

#### Single Word-Picture Matching

Participants viewed a picture (e.g., a crown) while simultaneously hearing a question about the identity of the picture in the form of “Is this a ___?” (Martin et al., 1999). The question included either the correct name (e.g., crown), a phonologically related word (e.g., clown), a semantically related word (e.g., hat), or an unrelated word (e.g., duck). There were 54 trials of each type. Participants responded “yes” to indicate that the picture matched the spoken word and “no” to indicate that it did not. Responses could be either verbal or nonverbal (pointing to the word yes/no or shaking/nodding the head). To index a person’s phonological processing, d’ values were calculated for a person’s ability to discriminate between the correct word and phonologically related distractors.^3^

#### Consonant Discrimination

Participants heard two consonants and indicated whether the consonant pair was the same or different (Martin and Breedin, 1992). When a consonant pair was different, the items varied by a single distinctive feature (e.g., ga, ba; ka, ga). There were two blocks of trials with 54 items each. Within a block, there were 22 matching and 32 non-matching trials. In the first block, the items were in consonant-vowel form; in the second block, items were in vowel-consonant form. Participants responded with a button press, and the dependent measure was percent correct responses.

#### Auditory Lexical Decision

Participants heard a stimulus and were asked to judge whether it was a real word (Martin and Breedin, 1992). There were 120 trials total, and half included real words. Nonwords were created by altering the initial or final phoneme of each word by a single phonetic feature (e.g., pickle, bickle). Of the 60 words, half were one syllable, and the other half were two syllables. Responses were made with a button press.

### Semantic Processing

#### Single Word-Picture Matching

A description of the single word-picture matching task, including a description of the semantic distractor condition, was presented in section Single Word-Picture Matching. To index a person’s semantic processing, d’ values were calculated for a person’s ability to discriminate between the target word and semantically related distractors.

#### Pyramids and Palm Trees

We used the picture subtest of the Pyramids and Palm Trees test (Howard and Patterson, 1992). Participants saw three pictures in a match-to-sample format and were asked to point to the picture that was most closely associated with the sample. There were 52 trials, and percent correct responses were the dependent variable.

### Sentence Comprehension

#### Active-Passive Comprehension

**Figure 2:**
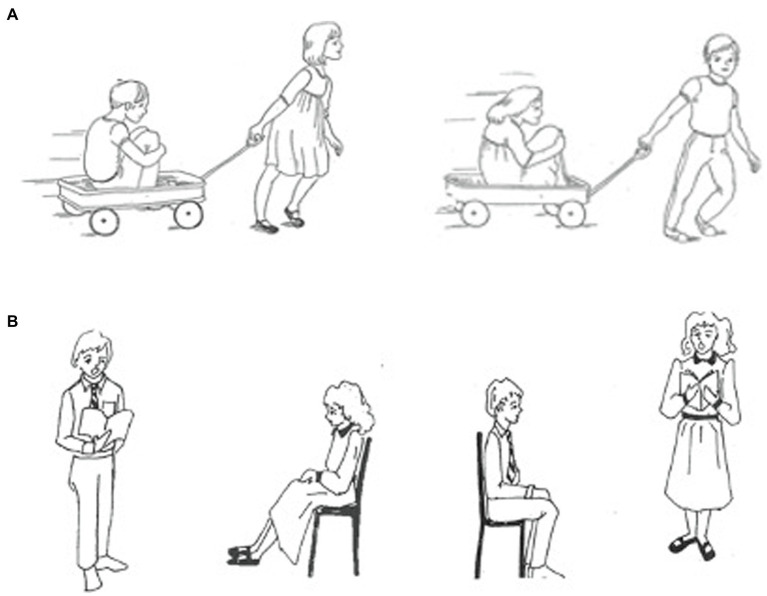
For reversal trials for the transitive sentences in the active-passive sentence comprehension task, the incorrect picture in a set presented the incorrect (i.e., reversed) action clause. For example, “The boy pulled the girl” was presented with a picture set in which the incorrect picture portrayed the girl pulling the boy **(A)**. For reversal trials for the dative sentences, the incorrect picture depicted the reversal of the relationship between the agent and the indirect object. For example, “The boy read a book to the girl” was presented with a picture set where the incorrect picture portrayed the girl reading a book to the boy **(B)**.

The active-passive comprehension task was first described by Martin (1987). In the task, participants listened to a sentence and chose from two pictures the one that matched the sentence. There were 56 sentences total, all with reversible relationships between the agent and object of the verb. Example sentences from each trial type are presented in Table 3. On 16 of the trials (called “reversal trials”), the incorrect picture showed the reversal of the agent and object, requiring the participant to use the syntactic information in the sentence to choose the picture showing the correct meaning relationship (Figure 2A). Half of the 16 sentences were in active form and the other half were in passive form. On 24 trials (called “lexical substitution trials”), the incorrect picture depicted either the incorrect agent, object, or action. There were eight trials (half passive and half active) for each lexical substitution type. On the remaining 16 trials, the sentences were in the dative case, and the incorrect picture depicted a reversal of the relationship between the agent and the indirect object (Figure 2B). Half of the dative sentences included a passive clause, and the other half included an active clause.

#### Relative Clause Comprehension

**Figure 3:**
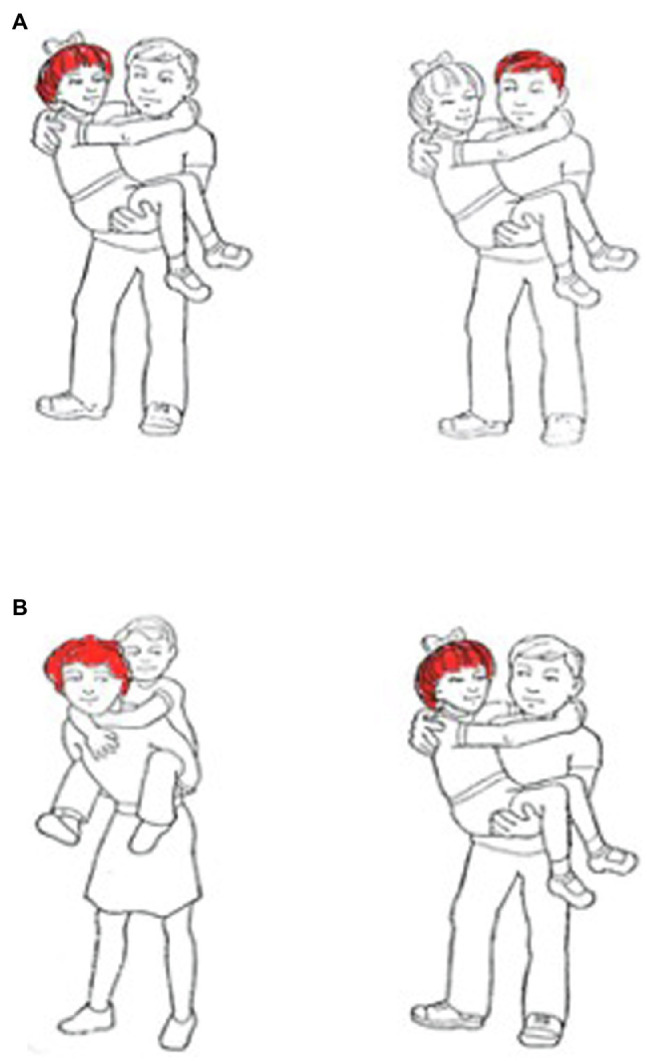
Each sentence in the relative clause sentence comprehension task was presented twice, each time with a different set of picture choices. For example, “The girl that the boy carried had red hair” was presented with a picture set in which the incorrect picture portrayed the correct action clause but the incorrect descriptive clause **(A)** and a set in which the incorrect picture portrayed the correct descriptive clause but the reversed action clause **(B)**.

The relative clause sentence comprehension task was also first described in Martin (1987). In the task, participants heard a sentence and chose from two pictures the one that matched the sentence. There were five sentence types (Table 1), each containing an action phrase and a descriptive phrase. Sentence types 1–4 were subject relative sentences where the head noun (in the example in Table 1, “boy”) was the subject of the embedded clause. Type 5 sentences were in the object relative form, where the head noun was the object of the embedded clause. Additionally, in sentence types 1 and 2, both the action and descriptive clauses were in the active form, differing only in which clause was embedded. In sentence types 3 and 4, the action clause was in passive form, again, differing only in which clause was embedded. Twelve sets of five sentences were created. Within each set, the same content words were used to create all five sentence types. Each sentence was presented twice, resulting in a total of 120 trials. On one presentation, the incorrect item in the picture pair depicted the correct action clause but the incorrect descriptive clause (Figure 3A). On the other presentation, the incorrect item in the picture pair depicted the correct descriptive clause but a reversal of the action clause (Figure 3B). Thus, when participants were asked to point to the picture that corresponded to the sentence that they heard, they had to use syntactic information in the sentences to choose the picture that portrayed the correct meaning relationships. A later version of this task included 12 lexical distractor sentences where in the incorrect picture, the noun, verb, or adjective was inaccurate (e.g., the hair was colored black rather than red). Nine participants did not complete the version with lexical distractor trials.

### Analysis

We used a case series approach to examine the relationship between sentence comprehension performance (as measured by either the relative clause sentence comprehension task or the active-passive comprehension task) and WM. Because there is a correlation between semantic and phonological WM, we used multiple regression to isolate the independent contribution of either semantic or phonological WM to sentence comprehension performance. We regressed performance on the more difficult sentence comprehension trial type of some contrast (e.g., object relative comprehension) on the less difficult comparison sentence type (e.g., subject relative comprehension), semantic WM, phonological WM, semantic single word processing, and phonological single word processing. In multiple regression, the independent contribution of each predictor variable is indicated by the significance of the predictor’s co-efficient (i.e., beta weight) independent of the other predictors in the model (Darlington, 1990). For example, a significant co-efficient for semantic WM in the model would indicate the significance of the contribution of semantic WM to predicting sentence comprehension in the more difficult condition independent of comprehension of the less difficult comparison sentence type, phonological WM, and semantic and phonological single word processing.

We screened for outliers in the multiple regression models by identifying any observations with both a studentized residual of more than 2.5 and a Cook’s d value that was greater than 3 standard deviations from the mean. In the relative clause sentence comprehension, one such outlier was removed from the contrast comparing performance on all passive sentence types to performance on all active sentence types. In the active-passive sentence comprehension, one outlier was removed from each of the three contrasts.

For phonological WM, semantic single word processing, and phonological single word processing, we created composite scores which encompassed performance on all tasks chosen to tap into participants’ processing in each of these three domains. Composite scores were computed by determining the first principal component factor scores for the relevant phonological WM, semantic processing, and phonological processing measures.^4^ Imputation was used if participants were missing one of the scores (*N* = 8 for phonological WM; *N* = 6 for semantic and phonological single word processing). The phonological WM composite included digit matching span and digit span. The semantic composite included the d’ semantic measure from single word-picture matching and the proportion correct on the Pyramids and Palm Trees (PPT). The phonological processing composite included the d’ phonological measure from single word-picture matching, the proportion correct on the consonant discrimination task, and the proportion correct on the auditory lexical decision task.

## Results

Coefficients and significance levels for all terms in all multiple regression models tested are described in Supplementary Table 3. In the results below, we focus on the independent contributions of semantic and phonological WM to active-passive and relative clause sentence comprehension performance.

### Active-Passive Sentence Comprehension

**Figure 4:**
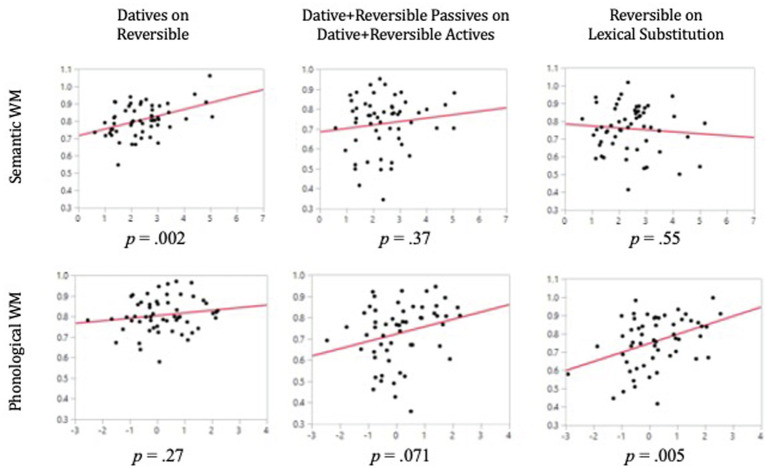
Effect leverage plots for the individual contributions of semantic and phonological WM in three models predicting performance on the active-passive sentence comprehension task. In all plots, the *Y*-axis represents sentence comprehension performance after all other effects included in the model have been factored out. The *X*-axis represents WM performance (semantic WM along the top row and phonological WM along the bottom) after all other effects included in the model have been factored out.

Mean performance on all sentence types in the active-passive comprehension task is presented in Table 5. As can be seen there, mean proportion correct for different conditions varied from a low of 0.70 for transitive passives to a high of 0.94 for lexical distractors. As expected, better performance was seen for active than passive structures. Somewhat unexpectedly, the means for the dative sentences were slightly higher than for the transitives. The multiple regression results for the active-passive sentence comprehension can be seen in Table 6. The four columns report statistics for the independent contributions of semantic and phonological WM for each contrast. For each of the three contrasts, the whole model was highly significant (all *p*’s < 0.0001). Note that the matched, baseline comprehension condition was included as a predictor in each model, driving the high level of overall significance for each model. Effect leverage plots for the effects of semantic and phonological WM (that is, plots showing the influence of these variables when controlling for the other variables) in each model can be found in Figure 4.

**Table 6:** Coefficients and significance levels for the independent contributions of semantic and phonological WM to active-passive sentence comprehension.

Contrast	*t*	Beta	*SE*	*p*
**Dative on Transitive**				
Semantic WM	3.27	0.038	0.012	0.002^*^
Phonological WM	1.13	0.013	0.011	0.27
**Dative + Transitive passives on** **Dative + Transitive actives**				
Semantic WM	0.90	0.017	0.019	0.37
Phonological WM	1.85	0.035	0.019	0.071
**Reversible mean on lexical distractors**				
Semantic WM	−0.60	−0.011	0.018	0.55
Phonological WM	2.97	0.050	0.017	0.005^*^

* *Indicates significance at p < 0.05*.

#### Reversible Datives on Transitives

When regressing the mean comprehension of dative sentence types on the mean for the reversible sentence types, semantic WM had a significant independent contribution [*b* = 0.038, *t*(54) = 3.27, *p* = 0.002], whereas the phonological WM composite did not [*b* = 0.013, *t*(54) = 1.13, *p* = 0.266]. Semantic processing also significantly predicted comprehension [*b* = 0.027, *t*(54) = 2.09, *p* = 0.042].

#### Dative and Transitive Passives on Dative and Transitive Actives

When mean comprehension of the dative and transitive passive sentences was regressed on the mean for dative and transitive active sentences, none of the predictor variables had a significant weight, but two were marginal (Supplementary Table 3).

#### Reversible Mean on Lexical Substitutions

When regressing comprehension of the mean of the reversible sentence trials on the trials with lexical substitutions, the phonological WM measure had a significant independent contribution [*b* = 0.050, *t*(54) = 2.97, *p* = 0.005] while semantic WM did not [*b* = −0.011, *t*(54) = −0.60, *p* = 0.552]. Additionally, in this model, semantic processing had a significant independent contribution to comprehension [*b* = 0.077, *t*(54) = 2.94, *p* = 0.005].

### Relative Clause Sentence Comprehension

**Figure 5:**
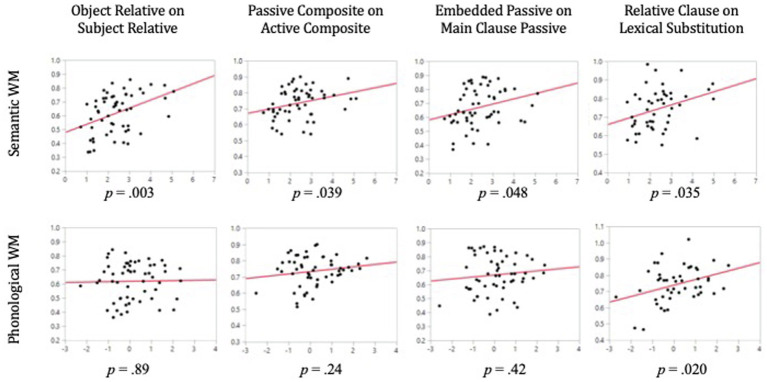
Effect leverage plots for the individual contributions of semantic and phonological WM in four models predicting performance on the relative clause sentence comprehension task. In all plots, the *Y*-axis represents sentence comprehension performance after all other effects included in the model have been factored out. The *X*-axis represents WM performance (semantic WM along the top row and phonological WM along the bottom) after all other effects included in the model have been factored out.

Mean performance for all sentence types in the relative clause comprehension task is presented in Table 7. The results for the multiple regression models predicting relative clause sentence comprehension can be seen in Table 8. The four columns report statistics for the independent contributions of semantic and phonological WM for each contrast. Effect leverage plots for the effects of semantic and phonological WM in each model can be found in Figure 5. As was done for the active-passive sentence types, we regressed the more difficult sentence type on the easier baseline sentence and on semantic WM, phonological WM, and the phonological and semantic processing measures.

**Table 7:** Descriptive statistics for performance on each trial type in the relative clause sentence comprehension task (*N* = 67, except lexical substitutions *N* = 47).

	Mean	Standard deviation	Range
(1) Active main clause	0.84	0.18	0.33–1
(2) Active embedded clause (subject relative)	0.79	0.19	0.42–1
(3) Passive main clause	0.80	0.21	0.29–1
(4) Passive embedded clause	0.67	0.20	0.25–1
(5) Active object relative	0.62	0.21	0.25–1
Lexical substitutions	0.92	0.09	0.67–1
**Combined conditions**			
Active mean	0.81	0.17	0.38–1
Passive mean	0.73	0.19	0.36–1
Relative clause mean (1–5)	0.74	0.17	0.40–1

**Table 8:** Coefficients and significance levels for the independent contributions of semantic and phonological WM to relative clause sentence comprehension.

Contrast	*t*	Beta	*SE*	*p*
**Object relative (5) on subject relative (2)**				
Semantic WM	3.16	0.059	0.019	0.003^*^
Phonological WM	0.14	0.002	0.018	0.89
**Passives (3 + 4) on actives (1 + 2)**				
Semantic WM	2.12	0.027	0.013	0.039^*^
Phonological WM	1.20	0.015	0.012	0.24
**Embedded passive (4) on main clause passive (3)**				
Semantic WM	2.03	0.038	0.019	0.048^*^
Phonological WM	0.82	0.015	0.018	0.42
**Mean of relative clause on lexical distractors**				
Semantic WM	2.19	0.035	0.016	0.035^*^
Phonological WM	2.42	0.035	0.014	0.020^*^

* *Indicates significance at p < 0.05*.

#### Object Relative on Subject Relative

In the regression of comprehension of type 5 sentences (object relatives) on type 2 sentences (subject relatives), semantic WM had a significant independent contribution to comprehension of object relative clause sentences [*b* = 0.059, *t*(55) = 3.16, *p* = 0.003], whereas phonological WM did not [*b* = 0.002, *t*(55) = 0.14, *p* = 0.887].

#### Passive on Active

In the regression of comprehension of the sentence types containing a passive (types 3 and 4) on matched sentence structures containing an active (types 1 and 2), the semantic WM measure had a significant independent contribution [*b* = 0.027, *t*(54) = 2.12, *p* = 0.039], whereas the phonological WM composite did not [*b* = 0.016, *t*(54) = 1.20, *p* = 0.236].

#### Embedded Passive on Main Clause Passive

In the regression of comprehension of sentences with an embedded passive (type 4) on the sentences with a main clause passive (type 3), semantic WM had a significant independent contribution in predicting comprehension [*b* = 0.038, *t*(55) = 2.03, *p* = 0.048], whereas the phonological WM measure did not [*b* = 0.015, *t*(55) = 0.82, *p* = 0.416].

#### Relative Clause Mean on Lexical Distractors

In the regression of mean comprehension across all sentence types (types 1–5) with reversal pictures on all sentence trials with lexical distractors, the semantic WM measure had a significant contribution [*b* = 0.035, *t*(46) = 2.19, *p* = 0.035] as did the phonological WM measure [*b* = 0.035, *t*(46) = 2.42, *p* = 0.020]. Additionally, semantic processing had a marginally significant contribution [*b* = 0.031, *t*(46) = 1.92, *p* = 0.062].

## Discussion

This work represents the first case series analysis of the relation between sentence comprehension (both relative clause sentence comprehension and active-passive comprehension) and semantic vs. phonological WM while controlling for single word processing. A summary of the results for each sentence comprehension contrast is presented in Table 9. We predicted that semantic WM, but not phonological WM, would have a significant independent contribution to the comprehension of sentences that theoretically place a higher demand on WM. This prediction was based on prior work which varied the distance over which words were integrated (Martin and Romani, 1994; Martin and He, 2004; Hamilton et al., 2009) and studies that manipulated semantic and syntactic interference (Tan et al., 2017; Tan and Martin, 2018). These studies generally found that semantic WM, but not phonological WM, predicted sentence comprehension for healthy young adults as well as people with brain damage.

**Table 9:** Summary of WM contributions to comprehension of target sentence when controlling for performance on baseline sentence.

Target sentence	Baseline sentence	Semantic WM?	Phonological WM?
**Active-Passive Sentence Comprehension**			
**Datives:** The boy read a book to the girl. The book was read to the girl by the boy.	**Transitives:** The boy pulled the girl. The girl was pulled by the boy.	•	
**Dative + Transitive Actives:** The boy read a book to the girl. They boy pulled the girl.	**Dative + Transitive Passives:** The book was read to the girl by the boy. The girl was pulled by the boy.		
**All Active-Passive Trials with Reversible images**	**Lexical distractor trials**		•
**Relative Clause Sentence Comprehension**			
**Object relative:** The boy that the girl carried had red hair.	**Subject relative:** The boy that carried the girl had red hair.	•	
**Passives:** The boy that had red hair was carried by the girl. The boy that was carried by the girl had red hair.	**Actives:** The boy that had red hair carried the girl. The boy that carried the girl had red hair.	•	
**Embedded Passive:** The boy that was carried by the girl had red hair.	**Main clause passive:** The boy that had red hair was carried by the girl.	•	
**All relative clause trials with reversible images**	**Lexical distractor trials**	•	•

*Examples of each sentence type are provided*.

From the active-passive comprehension task, we tested three comprehension contrasts for relations with semantic vs. phonological WM. For one of the three contrasts, our predictions were confirmed. When we regressed comprehension of dative sentences on the comprehension of reversible transitive sentences, we observed a significant weight for semantic WM, but not phonological WM. Given that the dative sentence types required the integration of three nouns while the transitive sentences only included two nouns, this result suggests a role for semantic WM in comprehending sentences when the number of propositions that must be maintained increases (Caplan and Waters, 1999).

When we regressed the mean of reversible transitive and dative sentences containing a passive on the mean of reversible transitive and dative sentences containing only active clauses, we did not observe a significant weight for either WM type. This finding was somewhat surprising considering passive structures are considered more difficult to comprehend in terms of assigning role relations, and thus, we predicted should place higher demands on WM. At the same time, the passive transitive sentences were very simple (e.g., “the boy was pushed by the girl”) and less likely to place demands on WM than the dative structures (e.g., “The boy was given a book by the girl”). This null result may be explained by the simplicity of the transitive sentences and by the inclusion of the dative structures in both the target and control conditions of this contrast, canceling out semantic WM’s role as an independent predictor of sentence comprehension. Finally, contrary to our predictions, when we regressed all sentences with reverse role pictures on all trials with lexical distractors, we only observed a significant effect of phonological WM. Again, the inclusion of the active and passive transitive sentence types in the mean may have diluted the effect of semantic WM that was observed for the dative sentences. We shall return to this issue of the source of significant effects when contrasting all sentence types with reversal distractors with those with lexical distractors after discussing the relative clause sentence results.

More straightforward evidence for semantic WM’s role in sentence comprehension comes from the relative clause comprehension results, where word meanings had to be retained across several intervening words (e.g., Gibson, 1998) and where there was the potential for semantic interference during cue-based retrieval (Tan and Martin, 2018). For three of the four contrasts from the relative clause sentence comprehension task, our predictions were confirmed. The first contrast of the object relatives vs. subject relatives showed a highly significant weight for semantic WM and an effect for phonological WM that was far from significance. These results suggest an important role for semantic WM in maintaining the meaning of the head noun across the interfering embedded clause subject noun to integrate with the main clause verb.

The next two contrasts that we tested from the relative clause comprehension task were motivated on the grounds that passive clause processing is slower and more prone to error in terms of the assignment of role relations than active clause processing (Ferreira et al., 2002). Thus, WM resources are taxed because information must be maintained for longer while this more difficult processing is carried out (Barrouillet and Camos, 2021). Both the contrast of the mean of main clause and embedded passives vs. mean clause and embedded actives as well as the contrast of the embedded passives vs. the main clause passives also indicated a significant role for the ability to maintain semantic representations. In these cases, semantic WM could be playing a role in maintaining semantic representations of the head nouns prior to integration with the main clause and embedded clause verbs. The role of semantic WM in the embedded passive vs. main clause passive contrast is consistent with the idea that processing an embedded clause is generally more difficult because it results in a longer period over which information must be maintained. One may have predicted that phonological WM, in addition to semantic WM, would have been significant in these contrasts: the word order for the agent and patient in passives is unusual, and phonological WM is often argued to be important for maintaining order information. However, as argued by McElree et al. (2003), retention of serial order does not seem to be critical in sentence processing. Instead, what is critical is maintaining sentence elements’ roles as they are derived. For example, in the type 4 sentences, listeners may have assumed that the head noun (e.g., the boy) would be the agent of the main clause and the embedded clause. However, when processing the embedded passive structure (e.g., was carried by), this assumption must be revised. Once the revision is complete, the object of the embedded passive can be interpreted as the original agent when it is processed.

Notably, in the final contrast for the relative clause sentence comprehension task where we regressed the mean comprehension performance for all relative clause sentences with reversal distractor pictures versus comprehension for those with lexical distractor pictures, both semantic WM and phonological WM predicted comprehension performance. While these results do provide support for the claim that semantic WM plays a critical role in relative clause sentence comprehension, the relationship with phonological WM was unexpected. Some have suggested that phonological WM may play a backup role in sentence comprehension, allowing for a review to check for the correct sentence interpretation (e.g., Miyake et al., 1994). One might have expected such a review to be more likely for the more complex and uncommon structures such as object relatives and passives. However, for the specific contrasts of more vs. less complex structures, the phonological WM weight did not approach significance. The comparison of performance on trials with reversal distractor pictures vs. lexical distractor pictures contrasts trials where deriving the syntactic structure is necessary to accurately perform the task compared to trials where it is not. These results suggest that the role of phonological WM was equivalent across sentences with differing demands deriving from the complexity of their structure.

Based on this finding, perhaps the tendency to use phonological WM as a backup record to check the result of comprehension occurs equally across all sentence types because all the sentence types required the integration of nouns, verbs, and adjectives not required in the lexical distractor condition. This tendency may be particularly strong in cases where, as in the current study, thematic role assignments can be reversed based solely on semantic factors and syntactic structure must be used to make the correct role assignments. For even the simplest type 1 sentences (e.g., the boy that had red hair carried the girl), either the boy or girl could plausibly be the agent or patient of the verb carry, and either could have red hair. For sentences without the possibility of role reversals (e.g., the apple that the boy ate was red), there may be no such tendency to carry out a comprehension check using a verbatim backup representation stored in phonological WM. Only future research could address questions regarding when exactly a phonological record plays a role in sentence comprehension. We observed some preliminary evidence in this regard when examining performance for only those participants who showed reasonably good comprehension on the reversible transitive and dative sentences—that is, obtaining a proportion correct of 0.70 or better (*N* = 25). For these individuals, the three contrasts of object on subject relatives, passives on actives, and embedded passives on main clause passives replicated the findings for the whole group in that all showed significant weights for semantic WM but not for phonological WM. Moreover, the contrast of trials with reverse role distractors and those with lexical distractors showed a marginally significant weight for semantic WM (*p* = 0.08) and a weight that was far from significance for phonological WM (*p* = 0.89) (See section 4 of Supplementary Materials). Thus, these findings provide at least some support for the suggestion that when syntactic processing is relatively preserved, there is little need to rely on a phonological WM backup representation. When syntactic processing is slowed or error-prone, individuals may need to retain a phonological representation downstream from the point of current processing. Subsequent experimental work will be needed to directly address this issue.

### Limitations

The present study provided evidence that WM capacities—as measured by recall of random digit or word lists—related to our participants’ ability to understand sentences, with semantic WM relating to the comprehension of more complex sentences relative to simpler sentences. As noted earlier, with the materials used here, we could not distinguish whether the source of difficulty was the distance across which integrations were made or due to interference from overlapping semantic features. Future research that assesses these two factors with the same individuals could determine whether independent deficits in the two could be uncovered.

Another limitation was that the nature of the role of phonological WM was unclear, as phonological WM capacity did not relate to the comprehension of more vs. less difficult sentences but to all sentence types. If phonological WM plays a backup role in sentence comprehension, then future work could address whether its role was more evident for individuals shown to have slowed lexical access or slowed syntactic processing for simple sentences.

Despite these limitations, the findings support the contention that general WM capacities, as tapped by the ability to retain random lists of digits and words, support comprehension. The study cannot, however, address whether other factors were also at play in participants’ degree of sentence comprehension impairment. It is possible that at least some of these individuals had difficulties in assigning syntactic structure *per se* that also impacted their comprehension, as has often been suggested in the literature (e.g., Caplan et al., 1988; Grillo, 2009). However, such approaches would not explain why we obtained our results relating semantic and phonological WM to comprehension if these syntactic deficits were the only source of comprehension deficits. That is, there is no straightforward way, for instance, to map between the capacities involved in maintaining the meanings of nouns from different semantic categories (as in our category probe task) and the kinds of features that might be encoded syntactically such as grammatical or thematic roles or inflections. We would also note that some studies of individuals with aphasia have found that while they may show decreased accuracy on tasks like picture matching, subtle online processing measures have sometimes suggested that they have processed structure correctly but appear to have lost that structural information when completing the comprehension task (e.g., Dickey and Thompson, 2009; Caplan et al., 2015), which might be associated with a WM deficit. Thus, further research is needed to address whether some individuals do have difficulties assigning syntactic structure beyond their WM deficits that might be revealed in online as well as end-of-sentence comprehension measures.

## Conclusion

The data reported here provide strong support for the role of semantic WM during sentence comprehension for sentence types argued to make heavy demands on WM relative to matched sentences with lesser demands. Phonological WM also played a role. However, the nature of phonological WM’s contribution is unclear, as it occurred across sentence types irrespective of structural demands. Future work is needed to further investigate the function of phonological WM across sentence types.

## Data Availability Statement

The raw data supporting the conclusions of this article will be made available by the authors, without undue reservation.

## Ethics Statement

The studies involving human participants were reviewed and approved by Rice University Institutional Review Board. The patients/participants provided their written informed consent to participate in this study.

## Author Contributions

RM, AH, and RZ: conception or design of the work and drafting the article. ON: data collection. RM, AH, ON, and RZ: data analysis and interpretation. All authors contributed to the article and approved the submitted version.

## Funding

This work was supported by the T.L.L. Temple Foundation Neuroplasticity Lab grant to Rice University and the National Science Foundation Research Experience for Undergraduates (REU) grant nos. (SMA-1853936 and SMA-1559393).

## Conflict of Interest

The authors declare that the research was conducted in the absence of any commercial or financial relationships that could be construed as a potential conflict of interest.

## Publisher’s Note

All claims expressed in this article are solely those of the authors and do not necessarily represent those of their affiliated organizations, or those of the publisher, the editors and the reviewers. Any product that may be evaluated in this article, or claim that may be made by its manufacturer, is not guaranteed or endorsed by the publisher.

## Footnotes

^1^It should be noted that for the structures in Table 1, it was not possible to separate out the effects of greater delayed integration and greater interference. For instance, when contrasting the type 5 object relatives with the type 2 subject relatives, there would be greater distance for integration for the object relatives as described in the text and greater interference because in the object relative there is interference from the other noun both when processing the embedded clause and main clause verbs, whereas in the subject relatives, there would only be interference when processing the main clause verb. For an investigation of comprehension when only interference is manipulated, see Tan and Martin (2018).

^2^Agrammatism was determined based on performance on a spontaneous speech sample scored using Quantitative Production Analysis (QPA; Rochon et al., 2000). Saffran et al. (1989) identified four measures from QPA as indicators of agrammatic speech. Those measures are the proportion of words produced that are in sentence utterances, the ratio of nouns to verbs produced, the production of closed class words, and the use of morphological inflection on the main verb. In our sample, 42 participants completed a spontaneous production task that was scored using QPA. Five participants fell within the range of agrammatic performance on at least three of the measures identified by Saffran et al. (1989).

^3^The measure of d’ is derived from signal detection theory and is calculated using a participant’s standardized hit rate (yes responses to correct trials) and their false alarm rates (incorrect yes response to distractor trials). In contrast to using proportion error for phonological and semantic distractor trials to index phonological and semantic processing, the use of phonological and semantic d’ measures for semantic and phonological processing also considers performance on correct trials (Stanislaw and Todorov, 1999). For example, a participant who had a 4 percent error rate on phonological distractor trials and performed at 93 percent on the correct trials would have a d’ phonological of 3.23, while another participant who also had a 4 percent error rate on phonological distractors but was 89 percent on the correct trials would have a d’ phonological of 2.98, indicating a weaker ability to distinguish targets from phonological distractors. In addition, we had the performance of 13 age matched control participants on single word single picture matching and could compare our participants ability to discriminate target and distractors to controls.

^4^Using the first principal component for a set of variables as a composite score gives similar results to creating a composite score by computing standard scores for each variable and averaging the standardized scores. Principal components analysis has an advantage over other methods of computing composite scores in that it provides specific weightings and allows for imputation of missing data. The phonological WM PCA had a eigenvalue of 1.75 and explained 87.47 percent of the variance in digit matching and digit span scores. The phonological single word processing PCA had an eigenvalue of 2.26 and explained 75.25 percent of the variance in the phonological d’, consonant discrimination, and auditory lexical decision scores. The semantic single word processing PCA had an eigenvalue of 1.51 and explained 75.56 percent of the variance in the semantic d’ and Pyramids and Palm Trees scores.
