# Supplementary material for: Semantic Working Memory Predicts Sentence Comprehension Performance: A Case Series Approach
Source: Front Psychol. 2022 Apr 29;13:887586. doi: 10.3389/fpsyg.2022.887586 (PMC9101950; doi:10.3389/fpsyg.2022.887586)
Supplement: Supplementary file 1 [file Data_Sheet_1.PDF]

Supplementary Material

1 Supplementary Table 1. Individual Western Aphasia Battery (WAB) and agrammatic classifications

**Participant**	Western Aphasia Battery (WAB) classification	Agrammatic (based on Saffran et al., 1989)
**BB**	Transcortical motor	+
**BQ**	Anomic	-
**BRH**	Anomic or conduction	-
**CA**	Anomic	-
**CB**	Anomic	-
**CV**	Conduction	-
**DZ**	Broca’s	
**DA**	Anomic	-
**DB**	Conduction	-
**DG**	Conduction	-
**DN**	Broca’s	
**DO**	Conduction	-
**DX**	Anomic	-
**EJ**	Broca’s	-
**ER**		-
**EV**	Anomic	-
**FK**	Transcortical motor	-
**FV**	Transcortical motor	+
**GV**	Conduction	-
**HZ**	Anomic	
**HA**	Broca's	
**HEQ**		
**HO**	Anomic or conduction	-
**HX**	Conduction	+
**KA**	Wernicke’s or conduction	-
**KG**	Anomic	-
**KI**		
**KK**	No Aphasia	-
**KLN**	Anomic	-
**KR**	Anomic	
**KS**	Broca's	+
**KV**	Broca’s or transcortical motor	
**LZ**	Anomic	-
**LB**	Anomic	-
**LQ**	Conduction	

**MB**	Anomic	-
**NA**	Broca's	
**NC**		
**NJ**	Conduction	-
**NK**		-
**NLA**	Conduction	-
**PB**	Broca's	+
**QO**	Anomic	-
**SE**	Anomic	-
**SH**	Anomic	-
**SI**	Broca's	
**SJ**	Anomic	-
**SQ**	Anomic	-
**SRX**	Anomic	
**SS**	Anomic	-
**SU**		-
**TG**	Anomic	-
**TQ**	Broca's or transcortical motor	-
**UO**	Broca's or anomic	-
**XB**	Anomic	-
**XF**	Broca's	

Note. Six participants did not complete the WAB and fourteen participants did not complete a spontaneous speech sample to be scored with QPA.

Supplementary Table 2. Individual participant performance on behavioral measures

	Working Memory			Single Word Processing					Active-Passive Sentence Comprehension					Relative Clause Sentence Comprehension					
**Participant**	Category Probe	Digit Matching Span	Digit Span	Semantic d'	Phonological d'	PPT	Consonant Discrimination	Auditory Lexical Decision	Reversible Active	Reversible Passive	Dative Active	Dative Passive	Lexical Substitutions	Type 1	Type 2	Type 3	Type 4	Type 5	Lexical Distractors
**BB**	1.50	4.50	3	2.53	3.76	0.88	0.94	0.93	0.63	0.38	0.75	0.50	0.88	0.54	0.46	0.42	0.33	0.38	
**BQ**	1.60	4.22	3	2.74	4.14	0.94	0.90	0.88	0.88	0.50	0.88	0.88	0.88	0.81	0.88	0.63	0.50	0.50	0.92
**BRH**	2	4.50	3	3.10	3.67	0.92	0.86		1	0.88	1	0.75	0.96	0.96	0.54	0.96	0.67	0.33	1
**CA**	4.57	6.50	8.5	2.04	3.22	0.83	0.98	0.87	1	1	1	1	1	1	0.96	1	0.96	1	1
**CB**	2.30	4	4.5	3.65	4.14	0.92	0.79	0.84	0.88	0.75	1	1	1	0.92	0.88	0.83	0.58	0.79	0.94
**CV**	1.33	2.30	2	3.10	2.93	0.92	0.58	0.69	0.63	0.50	0.38	0.63	0.88	0.67	0.46	0.29	0.42	0.54	
**DZ**	0.83	3.71		1.60	3.34	0.81	0.85	0.56	0.50	0.38	0.63	0.63	0.63	0.50	0.54	0.42	0.38	0.54	0.72
**DA**	2	6.44	6	3.21	3.76	0.94	0.93	0.85	1	1	1	0.88	1	1	0.92	0.96	0.75	0.67	

**DB**	1	3		2.18	3.54	0.92			0.75	0.50	0.75	0.13	0.84	0.58	0.75	0.50	0.25	0.63	1
**DG**	2	3.65	2.5	1.97	3.97	0.9	0.97	0.92	0.50	0.75	0.75	0.38	0.92	0.58	0.58	0.71	0.42	0.33	0.72
**DN**	0.75	2.13	2.5	2.07	3.97	0.46	0.73	0.77	0.38	0.38	0.50	0.25	0.71	0.46	0.46	0.58	0.71	0.54	0.89
**DO**	0.83	3.71	2.5	2.93	3.97	0.98	0.68	0.73	0.88	0.50	0.88	0.50	0.96	0.67	0.67	0.67	0.63	0.58	0.67
**DX**	1.50	3.55	2.5	3.10	2.93	0.92	0.70	0.72	0.88	0.38	0.88	0.50	0.96	0.58	0.79	0.79	0.75	0.71	0.89
**EJ**	2.22	2.10	2	3.01	2.31	0.92	0.74	0.71	0.88	0.63	0.50	0.50	0.79	0.83	0.63	0.67	0.67	0.58	0.89
**ER**	2.56	3	4	3.47	3.97		0.96	0.93	1	1	0.88	1	0.96	1	0.96	1	0.96	0.88	
**EV**	2	5.72	5	2.85	3.67	0.92	0.92	0.89	0.88	0.75	1	0.75	0.96	1	0.92	0.96	0.75	0.83	0.94
**FK**	4	5.77	6	3.21	3.76	0.96	0.94	0.87	1	1	1	1	1	1	1	1	1	1	1
**FV**	1.20	3.42	4.5	2.53	3.76	0.73	0.76	0.81	0.50	0.50	0.38	0.63	0.84	0.67	0.50	0.63	0.54	0.38	0.89
**GV**	0.41	2		2.29	2.13	0.96	0.66	0.52	0.75	0.50	0.88	0.63	0.83	0.79	0.71	0.75	0.58	0.29	0.89
**HZ**	4	5.63	6	3.33	3.76	0.96	0.85	0.93	1	1	1	1	0.96	1	0.96	1	0.92	0.92	0.94
**HA**	0.83	4.14		1.93	3.10	0.94	0.79	0.70	0.50	0.75	0.88	0.75	0.96	0.79	0.50	0.50	0.58	0.42	0.89
**HEQ**	5	4.81	5.5	3.67	3.76		0.95	0.78	1	1	1	1	1	1	1	1	0.96	1	

**HO**	2	4.84	3	2.71	3.76	0.71	0.94	0.78	0.88	0.75	0.88	0.88	0.96	1	0.92	0.96	0.96	0.88	0.89
**HX**	2.46	4.80	4.5	2.47	3.97	0.98	0.84	0.64	1	0.63	1	0.88	0.96	1	0.88	0.96	0.92	0.75	1
**KA**	0.45	4.31	4	2.52	1.74	0.92	0.52	0.52	0.88	0.50	1	0.38	0.875	0.46	0.63	0.54	0.58	0.33	0.78
**KG**	5.79	6	5	2.04	3.15	0.88	0.96	0.93	0.38	0.75	1	1	0.96	0.88	1	0.96	0.83	0.83	0.83
**KI**	1.67	3.80	4.5	3.11	3.93	0.98	0.84	0.83	0.63	0.88	0.88	0.75	0.88	1	0.85	1	0.50	0.45	
**KK**	5	6.38	7	3.65	3.93	0.98	0.86	0.83	1	1	1	1	1	1	1	1	1	1	1
**KLN**	0.63	3.71	4	2.43	3.02	0.96	0.79	0.62	0.75	0.75	0.75	0.63	0.96	0.83	0.75	0.79	0.42	0.54	1
**KR**	2	3.76	2.5	2.78	3.76	0.94	0.88	0.68	1	1	1	1	0.96	1	0.92	1	0.96	0.92	1
**KS**	2	3	3.5	3.76	3.76	0.94	0.93	0.79	0.88	1	1	1	1	1	0.96	0.96	0.88	0.88	1
**KV**	1.67	0.56	3	1.74	3.76	0.81	0.84	0.86	0.63	0.50	0.38	0.63	0.88	0.33	0.42	0.46	0.33	0.46	0.94
**LZ**	1	3.83	4.5	2.60	3.26	0.98	0.85	0.66	1	0.88	0.75	0.88	0.96	0.88	0.79	0.88	0.63	0.42	0.94
**LB**	2.37	4.72	2.5	3.47	3.97	0.98	0.98	0.78	0.88	0.88	1	0.88	1	1	0.92	0.96	0.71	0.67	1
**LQ**	3.33	1.46	1	4.14	3.65	0.9	0.67	0.67	0.75	0.63	0.88	0.75	1	0.96	0.67	0.79	0.50	0.54	0.83
**MB**	2.71	3.33	3	3.84	3.84	1	0.93	0.73	1	0.88	1	0.75	1	1	1	0.96	0.96	0.75	1
**NA**	3.40	3.76	4	3.20	3.84	0.9	0.83	0.98	0.63	0.50	0.88	0.75	0.96	0.88	0.92	0.67	0.54	0.50	0.78

**NC**	3	5.08	3.5	3.86	3.76	0.96	0.91	0.86	0.88	1	0.75	1	0.88	1	0.88	1	0.63	0.75	
**NJ**	1.50	4.13	3.5	2.71	3.84	0.88	0.94	0.79	1	0.50	0.63	1	0.96	0.92	0.92	0.88	0.67	0.63	0.89
**NK**	1.50	3.60	3	3.67	3.76	0.94	0.96	0.89	1	1	0.88	0.88	1	0.86	1	0.74	0.57	0.47	
**NLA**	2	5.44	4.5	3.63	3.54	0.98	0.87	0.78	0.75	0.75	0.88	0.89	0.96	0.96	0.88	0.92	0.67	0.42	0.89
**PB**	1.40	2.36	3	1.76	3.07	0.79	0.77	0.70	0.75	0.25	0.88	0.38	0.84	0.75	0.58	0.33	0.42	0.25	0.89
**QO**	3.50	4.84	3.5	3.93	3.93	0.98	0.94	0.90	1	1	1	1	1	0.96	1	1	1	0.83	1
**SE**	3.14	4	4	2.52	3.84	0.98	0.97	0.82	0.88	0.50	1	0.75	1	0.92	0.75	0.83	0.42	0.46	1
**SH**	3	4	3	3.20	3.34	0.98	0.90	0.84	0.75	0.75	1	0.63	0.88	0.92	0.83	1	0.71	0.50	0.83
**SI**	0.50	1	1.5	1.75	2.80	0.42	0.57	0.53	0.88	0.75	1	0.25	0.55	0.58	0.71	0.63	0.50	0.63	0.89
**SJ**	2.38	4.40	2.5	3.34	3.84	0.98	0.86	0.87	0.75	0.50	0.88	0.63	0.96	0.88	0.71	0.71	0.75	0.58	1
**SQ**	3	6.50	4.5	3.67	3.76	0.9	0.76	0.91	1	0.88	0.75	0.88	0.96	0.79	0.92	1	0.58	0.67	1
**SRX**	3.67	4.59	5	3.38	3.93	0.94	0.87	0.82	1	1	1	0.88	1	0.92	0.96	0.88	0.71	0.67	1
**SS**	2.86	4.13	4.5	3.34	3.84	0.98	0.91	0.84	0.63	0.38	0.88	0.38	0.96	0.92	0.83	0.88	0.75	0.71	1
**SU**	3.76	4.81	5	2.71	3.76	1	0.96	0.94	1	1	1	1	1	1	1	0.88	0.92	0.71	1

**TG**	6.50	6.50	6.5	3.10	3.76	0.92	0.93	0.88	1	0.88	1	1	0.96	1	1	1	0.96	1	1
**TQ**	2	2.50	3	2.52	3.84	0.87	0.83	0.70	0.38	0.38	0.63	0.50	0.88	0.63	0.58	0.33	0.54	0.46	0.78
**UO**	4.46	4.14	3	3.21	3.67	0.96	0.83	0.88	0.63	0.50	0.88	0.88	1	0.79	0.46	0.83	0.58	0.54	1
**XB**	1.83	5.80	5	3.33	3.67	0.94	0.92	0.78	1	0.38	1	0.50	0.92	0.92	0.75	0.83	0.63	0.33	0.94
**XF**	3.33	3.67		2.16	3.76	0.88	0.94		0.63	0.38	0.88	0.63	0.88	0.83	0.54	0.79	0.54	0.29	

3 **Supplementary Table 3. Coefficients and significance levels for the independent contributions of semantic and phonological WM, semantic and phonological single word processing and baseline sentence comprehension to target sentence comprehension**

Contrast	t	Beta	*SE*	*p*
**Active/Passive Comprehension**				
**Dative on transitive**				
Semantic WM	3.27	0.038	0.012	.002*
Phonological WM	1.13	0.013	0.011	.27
Semantic single word processing	2.09	0.027	0.013	.042*
Phonological single word processing	-0.15	-0.002	0.011	.88
Transitive sentence comprehension	5.89	0.454	0.078	<.0001*
**Dative+Transitive Passives on Dative+Transitive Actives**				
Semantic WM	0.90	0.017	0.019	.37
Phonological WM	1.85	0.035	0.019	.071
Semantic single word processing	1.74	0.038	0.022	.088
Phonological single word processing	1.51	0.026	0.017	.14
Dative+Transitive actives sentence comprehension	3.23	0.492	0.152	.002*
**Reversible on Lexical Distractors**				
Semantic WM	-0.60	-0.011	0.018	.55
Phonological WM	2.97	0.050	0.017	.005*
Semantic single word processing	2.94	0.077	0.026	.005*
Phonological single word processing	0.35	0.006	0.017	.73
Lexical distractor sentence comprehension	1.82	0.639	0.351	.075
**Relative Clause Comprehension**				
**Object relative (5) on subject relative (2)**				
Semantic WM	3.16	0.059	0.019	.003*

Phonological WM	0.14	0.002	0.018	.89
Semantic single word processing	-1.77	-0.037	0.021	.084
Phonological single word processing	-0.79	-0.013	0.017	.44
Subject relative sentence comprehension	5.29	0.736	0.139	<.0001*
**Passives (3+4) on actives (1+2)**				
Semantic WM	2.12	0.027	0.013	.039*
Phonological WM	1.20	0.015	0.012	.24
Semantic single word processing	0.25	0.004	0.016	.80
Phonological single word processing	-1.26	-0.014	0.011	.21
Actives (1+2) sentence comprehension	7.49	0.861	0.115	<.0001*
**Embedded passive (4) on main clause passive (3)**				
Semantic WM	2.03	0.038	0.019	.048*
Phonological WM	0.82	0.015	0.018	.42
Semantic single word processing	-0.51	-0.011	0.021	.61
Phonological single word processing	-1.22	-0.020	0.016	.23
Main clause passive sentence comprehension	4.73	0.603	0.127	<.0001*
**Mean of relative clause on lexical distractors**				
Semantic WM	2.19	0.035	0.016	.035*
Phonological WM	2.42	0.035	0.014	.020*
Semantic single word processing	1.92	0.031	0.016	.062
Phonological single word processing	0.05	0.001	0.015	.96
Lexical distractors sentence comprehension	2.35	0.471	0.201	.024*

Note. *indicates significance at $p < .05$.

4 Reanalysis of Relative Clause Comprehension in High Performers

To investigate the potential backup role for phonological WM in sentence comprehension we examined the relative clause sentence contrasts in those participants who had high performance on the active-passive sentence comprehension task (above 0.7 proportion correct). Individual participant performance on the behavioral tasks can be seen in Supplementary Table 2. This criterion left 31 participants in the relative clause analyses, 25 of which had completed the lexical distractors trials. We tested the relationship between WM and relative clause comprehension in high performers to examine whether phonological WM was a significant predictor of relative clause sentence comprehension when only those with preserved syntactic processing were included in the model. Table 1 reports statistics for the independent contributions of semantic and phonological WM for each contrast. As in the original multiple regression models, we regressed the more difficult sentence type on the easier baseline sentence and on semantic WM, phonological WM, and the phonological and semantic processing measures.

4.1.1 Object Relative on Subject Relative

In this sample the regression of comprehension for type 5 sentences (object relatives) on type 2 sentences (subject relatives) matched the results showed the same pattern as the original analysis. Semantic WM showed a significant independent contribution to comprehension of object relative clause sentences ($b = 0.078$, $t\ (30) = 3.19$, $p = .004$), whereas phonological WM did not ($b = -0.007$, $t\ (30) = -0.03$, $p = .763$).

4.1.2 Passive on Active

The new regression of comprehension of the sentence types containing a passive (types 3 and 4) on sentence types containing an active (types 1 and 2) matched the pattern of results of the original analysis. The semantic WM measure had a significant independent contribution in predicting comprehension ($b = 0.036$, $t\ (30) = 2.55$, $p = .017$), whereas the phonological WM composite did not ($b = -0.009$, $t\ (30) = -0.70$, $p = .488$).

4.1.3 Embedded Passive on Main Clause Passive

In the new regression of comprehension of sentences with an embedded passive (type 4) on sentences with a main clause passive (type 3) the results again matched the pattern of the original analysis. Semantic WM had a significant independent contribution ($b = 0.082$, $t\ (30) = 3.40$, $p = .002$), whereas the phonological WM measure did not ($b = -0.037$, $t\ (30) = -1.55$, $p = .133$).

4.1.4 Relative Clause Mean on Lexical Distractors

When we regressed mean comprehension across all sentence types (types 1-5) with reversal pictures on all sentence trials with lexical distractors in this sample, the results differed from the original analysis. The semantic WM measure had a marginally significant contribution ($b = 0.030$, $t\ (24) = 1.82$, $p = .084$) while the phonological WM measure was not significant ($b = -0.002$, $t\ (24) = -0.14$, $p = .893$). Additionally, phonological processing has a significant contribution ($b = 0.058$, $t\ (24) = 2.79$, $p = .012$)

Supplementary Table 4. Coefficients and significance levels for the independent contributions of semantic and phonological WM to sentence comprehension in high performers

Contrast	t	Beta	*SE*	*p*
**Object relative (5) on subject relative (2)**				
Semantic WM	3.19	0.078	0.024	.004*
Phonological WM	-0.30	-0.007	0.023	.76
**Passives (3+4) on actives (1+2)**				
Semantic WM	2.55	0.036	0.014	.017*
Phonological WM	-0.70	-0.009	0.013	.49
**Embedded passive (4) on main clause passive (3)**				
Semantic WM	3.40	0.008	0.024	.002*
Phonological WM	-1.55	-0.037	0.024	.13
**Mean of relative clause on lexical distractors**				
Semantic WM	1.82	0.030	0.017	.084
Phonological WM	-0.14	-0.002	0.016	.89

Note. *indicates significance at $p < .05$.
